# Comparison of Anthraquinones, Iridoid Glycosides and Triterpenoids in *Morinda officinalis* and *Morinda citrifolia* Using UPLC/Q-TOF-MS and Multivariate Statistical Analysis

**DOI:** 10.3390/molecules25010160

**Published:** 2019-12-31

**Authors:** Maoyuan Wang, Qinglong Wang, Qing Yang, Xiaoxia Yan, Shixiu Feng, Zhunian Wang

**Affiliations:** 1 Tropical Crops Genetic Resources Institute, Chinese Academy of Tropical Agricultural Sciences/Key Laboratory of Crop Gene Resources and Germplasm Enhancement in Southern China, Ministry of Agriculture, Haikou 571101, China; wmy81@163.com (M.W.); qlwang1983@163.com (Q.W.); yangqinghnu@163.com (Q.Y.); yxx6232@163.com (X.Y.); 2 Tropical Wild Plant Gene Resource, Ministry of Agriculture, Danzhou 571737, China; 3 Key Laboratory of South Subtropical Plant Diversity, Fairy Lake Botanical Garden, Shenzhen & Chinese Academy of Sciences, Shenzhen 518004, China

**Keywords:** *Morinda officinalis*, *Morinda citrifolia*, UPLC/Q-TOF-MS, multivariate statistical analysis

## Abstract

Roots of *Morinda officinalis* and *Morinda citrifolia* have been interchangeably used in traditional Chinese medicine. However, there is no experimental evidence to support this. In this study, a ultra-performance liquid chromatography quadrupole time-of-flight mass spectrometry (UPLC/Q-TOF-MS)-based approach and a multivariate statistical analysis (MSA) were adopted to compare the difference in the chemical compounds present in the root extract of *M. officinalis* and *M. citrifolia*. There were 26 anthraquinones, 15 triterpenes, and 8 iridoid glycosides identified in the root extracts of *M. officinalis*, 30 anthraquinones, 1 triterpene, and 8 iridoid glycosides in the root extracts of *M. citrifolia*. Among these, 25 compounds presented in both plants. In addition, a principal component analysis (PCA) showed that these two herbs could be separated clearly. Furthermore, an orthogonal partial least squares-discriminant analysis (OPLS-DA) found 9 components that could be used as chemical markers to discrimination the root extracts of *M. officinalis* and *M. citrifolia*. In addition, the results of a Cell Counting Kit 8 (CCK-8) assay and cell colony formation assay indicated that methanol root extracts of *M. officinalis* and *M. citrifolia* showed no cell cytotoxicity to normal cells, even promoted the proliferation of normal liver cells. To our knowledge, this is the first time that the differences between the root extracts of *M. officinalis* and *M. citrifolia* (Hainan province) have been observed systematically at the chemistry level.

## 1. Introduction

*Morinda officinalis (M. officinalis)* and *Morinda citrifolia* (*M. citrifolia*) belong to the family Rubiaceae [1]. The roots of *M. officinalis* have long been used ass traditional medicine for the strengthening of bones and immunity, and the nourishment of the kidneys [2]. It has also been used to treat impotence, osteoporosis, depression and inflammatory diseases, such as rheumatoid arthritis and dermatitis [2]. Moreover, the clinical effects of *M. officinalis* have been proved by modern pharmacological studies [3,4,5,6]. The fruit of *M. citrifolia* is also called Noni in Hawaii and the juice made of it has been used as alternative medicine for arthritis, high blood pressure, diabetes, mental depression, sprains, and muscle aches [7,8,9]. The *M. citrifolia* fruit extract has been shown to exhibit antimicrobial, antifungal, antioxidant, anti-arthritic, and anticancer activities [10,11]. Furthermore, it also shows anxiolytic and sedative activities, and immunity enhancing activity [12,13]. *M. officinalis* and *M. citrifolia* were commonly distributed in the south of China (Guangdong, Guangxi and Hainan province) and are named as “HAIBAJI” and “BAJITIAN”. In Hainan province, both plants are surprisingly called BAJI, and their roots have been used as edible herbal plants to treat sexual impotence, spermatorrhea, irregular menstruation, and female infertility [14]. However, evidence for such uses remains elusive, although both plants contain anthraquinones and iridoid glycosides [1,15]. In this study, we aim to determine whether the roots of the two plants have the same active chemical constituents in terms of their pharmaceutical functions. Quadrupole time-of-flight mass spectrometry (Q/TOF-MS) coupled with Ultra-performance liquid chromatography (UPLC) can be comprehensively used to analyze unknown compounds in an organism [16]. Hence, this study examines the principal chemical constituents of the roots of both species using UPLC/QTOF-MS coupled with a multivariate statistical analysis, and subsequently the maker components were elucidated by a principal component analysis (PCA).

## 2. Results and Discussion

### 2.1. Identifying the Chemical Constituents of Morinda officinalis (M. officinalis) and Morinda citrifolia (M. citrifolia) Based on UPLC/Q-TOF-MS/MS

**Figure 1:**
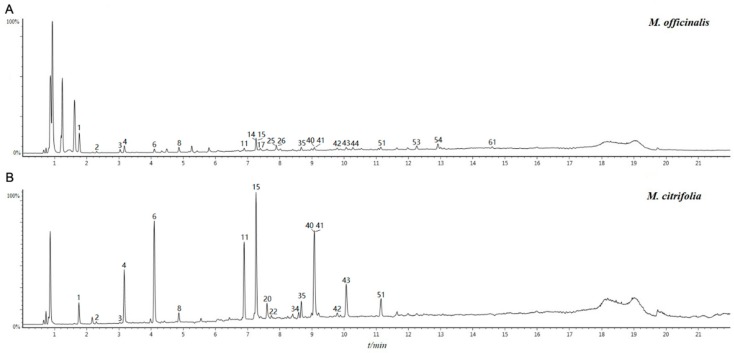
The total ion chromatogram (TIC) in the negative ion mode of samples: A for methanol extract of roots of *M. officinalis* roots, B for methanol extract *M. citrifoli* roots.

**Figure 2:**
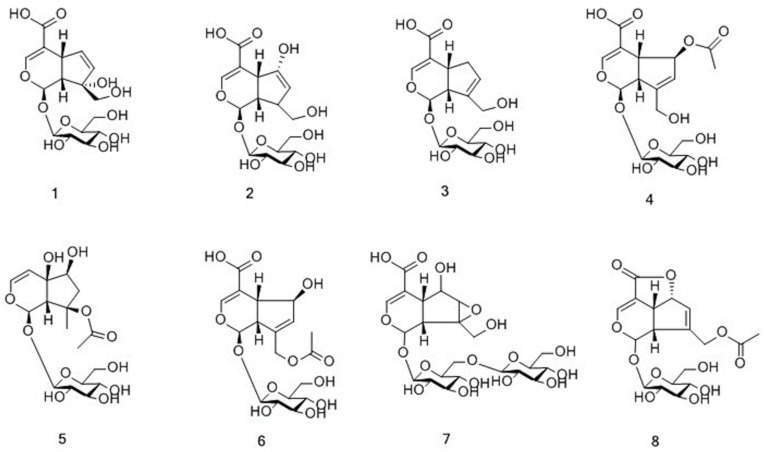
Chemical structures of iridoid glycosides identified in *M. citrifolia* and *M. officinalis.*

The total ion chromatogram (TIC) in the negative ion mode of *M. officinalis* and *M. citrifolia* root extracts is illustrated in Figure 1. The information of identified compounds is summarized in Table 1. Compounds **1**–**8** were identified as iridoid glycosides, and their structures are displayed in Figure 2. Generally, this class of compounds underwent a loss of a glucose unit (162) to yield a fragment (M-162) and a subsequent elimination of 44 (CO_2_) and/or 28 (CO) could occur after the loss of glucose unit (Compounds **1** and **3**). Compounds **5**, **6**, and **8** easily gave fragment ions of a glucoside (180), C_2_H_2_O (42), and COOH (45) produced from the deprotonated ion [17,18,19]. The proposed fragmentation pathway is shown in Figure S1.

**Figure 3:**
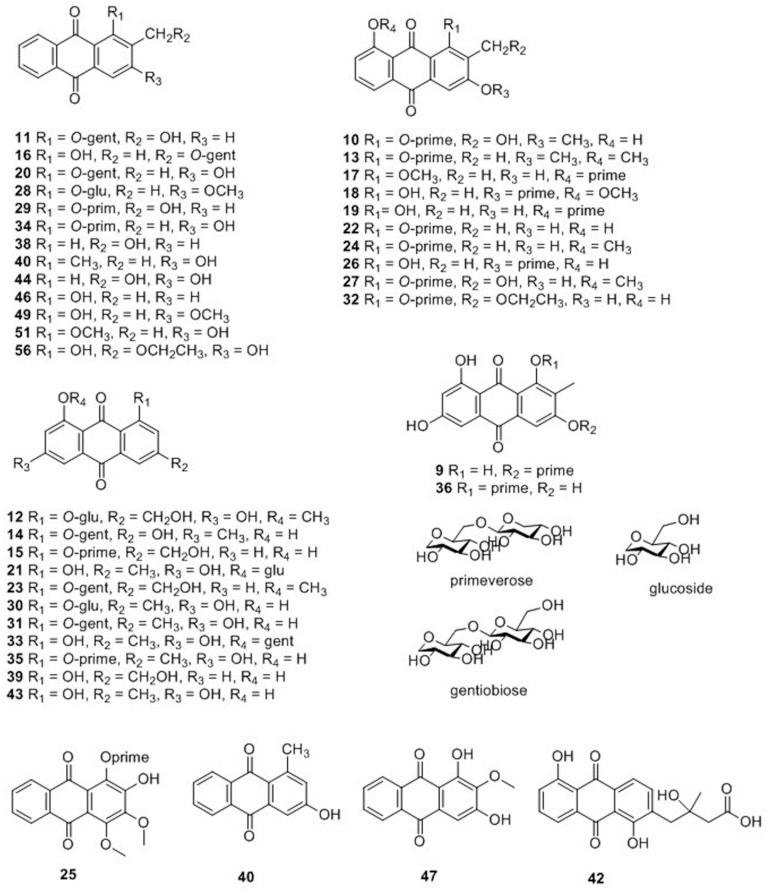
Chemical structures of anthraquinones identified in *M. citrifolia* and *M. officinalis.*

Compounds **9**–**36**, **38**–**44**, **46**–**47**, **49**, **51**, **56** were characterized as anthraquinones, and their structures are displayed in Figure 3. Based on whether they had a glycosyl group, the compounds were further classified into either anthraquinone glycosides (**9**–**36**, **41**) or anthraquinone aglycones (**38**–**40**, **42**–**44**, **46**–**47**, **49**, **51**, **56**). The parent ions of anthraquinone glycosides yielded fragment ions by effortlessly losing a glucose unit (162), xyloside unit (132), CH_2_ (14), and HCOOH (46) [17,18,19]. The proposed fragmentation pathway is shown in Figure S2. By contrast, anthraquinone aglycones were relatively stable, thus their fragments were difficult to determine.

**Figure 4:**
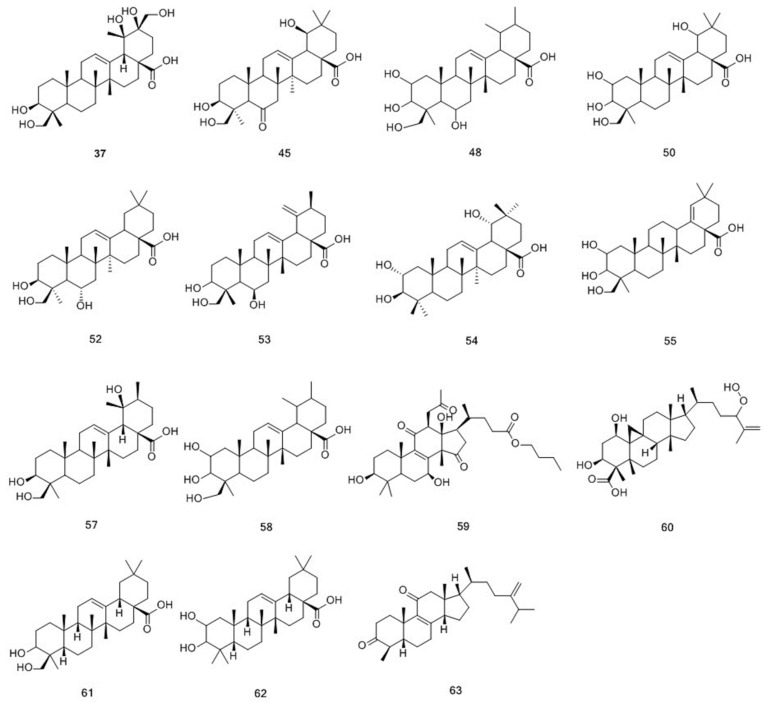
Chemical structures of triterpenoids identified in *M. citrifolia* and *M. officinalis.*

Compounds **37**, **45**, **48**, **50**, **52**–**55**, **57**–**63** were determined as triterpenes, and their structures are displayed in Figure 4 [20]. The structure of triterpenes is relatively stable, and it commonly formed ions that were produced by the loss of a unit of H_2_O. To date, approximately 100 compounds have been isolated from *M. officinalis,* which mainly are polysaccharides, oligosaccharides, anthraquinones, triterpenes, and iridoid glycosides [2]. Approximately, 160 compounds have been isolated and identified from *M. citrifolia,* which mainly comprise anthraquinones, organic acids, alkaloid, terpenes and volatile compounds [11]. In the present study, 49 compounds from the methanol extract of *M. officinalis* roots were identified, classified into 26 anthraquinones, 15 triterpenes, and 8 iridoid glycosides. At the same time, 39 compounds were identified from the methanol extract of *M. citrifolia* roots, including 30 anthraquinones, 1 triterpene, and 8 iridoid glycosides. Among them, 25 compounds were found to be present in both plants. These compounds are classified into 16 anthraquinones, 1 triterpene, and 8 iridoid glycosides. Detailed information on the identified compounds is shown in Table 1.

### 2.2. Tentative Identification of Potential Marker Compounds from M. officinalis and M. citrifolia

**Figure 5:**
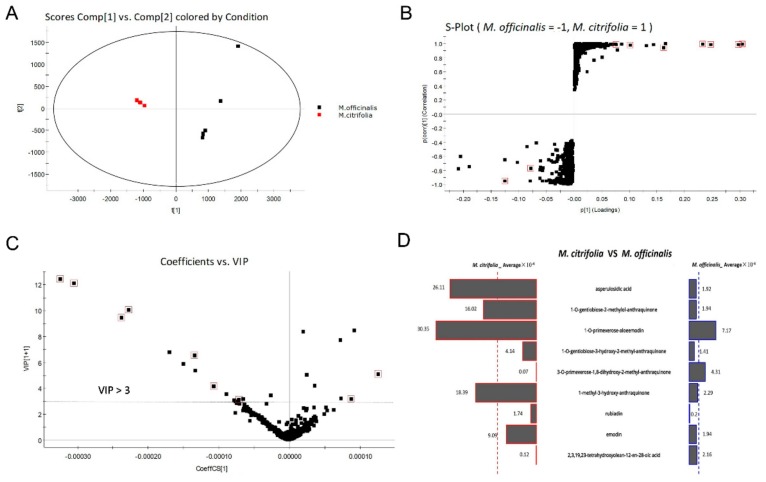
Selected potential biomarkers from *M. officinalis* and *M. citrifolia* based on principal component analysis (PCA) and orthogonal partial least squares-discriminant analysis (OPLS-DA). (**A**) PCA score plot; (**B**) S-Plot from OPLS-DA of *M. officinalis* and *M. citrifolia*. (**C**) The variable importance in projection (VIP) score of selected markers; (**D**) Butterfly shows the variable averages by group of selected potential marker compounds. The most differential compounds are marked with a red square.

To clarify the compound differences between the methanol extracts of *M. officinalis* and *M. citrifolia* roots, an untargeted metabolite profiling of the extracts was conducted and analyzed by UPLC-QTOF-MS/MS and Progenesis QI software. The PCA scores plot (Figure 5A) indicates that the chemical compositions of *M. officinalis* and *M. citrifolia* differ from one another. *M. officinalis* and *M. citrifolia* root samples were clearly distinguished by the principal component 1 (PC1). Furthermore, to explore the potential biomarkers corresponding to the differences between *M. officinalis* and *M. citrifolia* root extracts, UPLC-QTOF-MS/MS data were processed by a supervised OPLS-DA. Through an S-plot analysis at each point, the X- and *Y*-axis showed the variable contributions and sample correlations. The compounds with significant differences between groups were called marker compounds, and were plotted at the top right (1) and bottom left (-1). Therefore, nine ions (red box mark) were selected as candidates, to chemically distinguish *M. officinalis* from *M. citrifolia* root extracts (Figure 5B).

The variable importance in projection (VIP) plot (Figure 5C) showed that all the 7 selected potential marker compounds in *M. citrifolia* root extracts and 2 selected potential marker compounds in *M. officinalis* root extract have a high VIP value (VIP > 3), suggesting that these marker compounds are largely responsible for the chemical difference between these two samples. Furthermore, the variable average between groups clearly showed content discrepancy of the selected marker compounds in *M. citrifolia* and *M. officinalis* root extracts (Figure 5D). The 9 selected markers were asperulosidic acid (**6**), 1-*O*-gentiobiose-2-methylol-anthraquinone (**11**), 1-*O*-primeverose-aloe-emodin (**15**), 1-*O*-gentiobiose-3-hydroxy-2-methyl- anthraquinone (**20**), 3-*O*-primeverose-1,8-dihydroxy-2-methyl-anthraquinone (**26**), 1-methyl-3-hydroxy-anthraquinone (**40**), rubiadin (**41**), emodin (**43**), and 2,3,19,23-tetrahydroxyolean-12-en-28-oic acid (**50**). The results showed that the levels of 3-*O*-primeverose-1,8-dihydroxy-2-methyl-anthraquinone (**26**) and 2,3,19,23-tetrahydroxyolean-12-en-28-oic acid (**50**) were relatively higher in the root extract of *M. officinalis*, than in *M. citrifolia* (Figure 5D). The other seven compounds were relatively higher in *M. citrifolia* roots. Among them, compounds **11**, **15,** and **40** are anthraquinones, which are the characteristic compounds of the family Rubiaceae. Asperulosidic acid (**6,** iridoid glucosides) is one of the most common pharmacologically active ingredients in *M. officinalis* [2]. Previous reports found that it exhibited anti-inflammatory, anti-renal fibrosis, and antibacterial activities [21,22].

### 2.3. The Cytotoxicity of the Methanol Extracts of M. officinalis and M. citrifolia Roots

**Figure 6:**
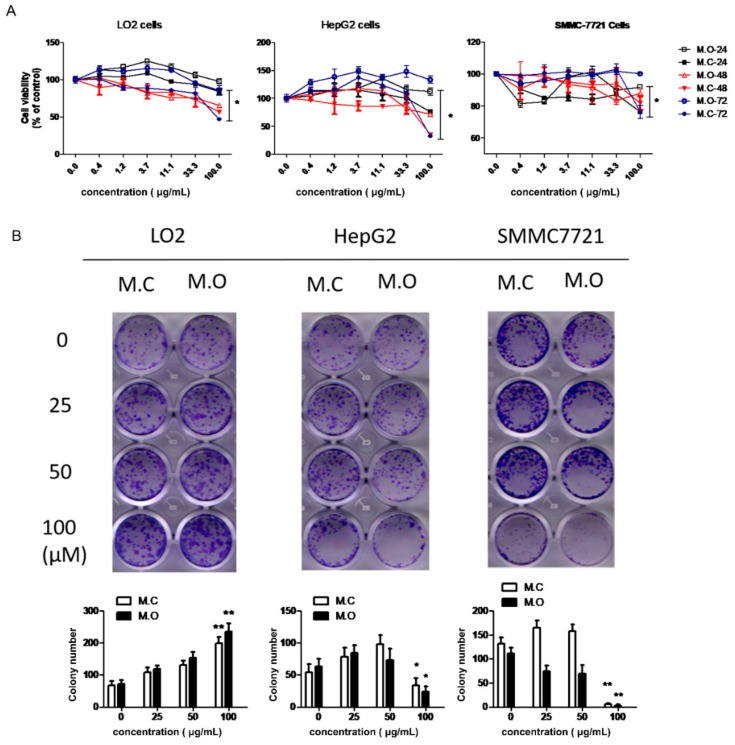
The effect of *M. officinalis* and *M. citrifolia* root extracts on cell growth. (**A**) Cell viability was evaluated by a CCK-8 assay kit. * *p* < 0.05, compared to 24 h treatment group. (**B**) The colony formation ability of cells was detected through a colony formation assay. * *p* < 0.05, ** *p* < 0.01, compared with untreated cells. M.C: the methanol extract from *M. citrifolia* root. M.O: the methanol extract from *M. officinalis* root.

To further investigate the differences in the methanol extracts of *M. officinalis* and *M. citrifolia* roots in terms of cytotoxicity, a CCK-8 assay was used to examine the cell viability of three liver cell lines (normal cells: LO2; cancer cells: HepG2, SMMCH771) after treatment by *M. officinalis* and *M. citrifolia* root extracts. As shown in Figure 6A, the two extracts were non-cytotoxic to the cells after a treatment of 24 h at concentrations of up to 100 μg/mL. When treated for 48 and 72 h, lower cell viability was observed in *M. citrifolia root extract* treatment groups compared to *M. officinalis root extract* treatment groups. In addition, a colony formation assay was used to evaluate the long-term impact of the *M. officinalis* and *M. citrifolia* root extracts on the cells. The results showed that a high dose of *M. officinalis* and *M. citrifolia* root extracts promoted the cell proliferation of LO2 cells, and inhibited the cell proliferation of HepG2 and SMMCH771 cells (Figure 6B). No significant differences were found between the root extracts of *M. officinalis* and *M. citrifolia* in terms of long-term impact on HepG2 and SMMCH771 cells. Hence, it is suggested that the methanol extracts of *M. officinalis* and *M. citrifolia* roots showed no cell cytotoxicity to normal cells, and even promoted the proliferation of normal liver cells. Meanwhile, compared to *M. officinalis root* extract, *M. citrifolia* root extracts exhibited stronger cell cytotoxicity to liver cancer cells.

Numerous studies indicated that *M. citrifolia* extracts exhibited anticancer activity [23]. For instance, the leaf extract of *M. citrifolia* has an inhibitory effect on lung cancer through strengthening immune response [24]. Its fruit extracts induced apoptosis and suppressed migration in human liver and breast cancer cells [25]. In contrast, few publications report the antitumor activity of *M. officinalis.* Our results are consistent with previous reports that *M. officinalis* root extracts show poor cell cytotoxicity toward cancer cells, compared with *M. citrifolia* root extracts.

## 3. Materials and Methods

### 3.1. Instruments and Materials

A Waters ACQUITY UPLC (Milford, MA, USA) coupled to a Xevo G2-XS Q-Tof (Milford, MA, USA) mass spectrometer was utilized. The ACQUITY UPLC HSS T3 RP-18 column (100 × 2.1 mm, 1.8 μm) was purchased from Waters (Milford, MA, USA). Masslynx V4.1, UNIFI^®^ and Progenesis QI were also from Waters (Milford, MA, USA). A precision scale (Secura513-1CN) was used (Sartorius Corporation, Germany).

Pall GHPAcrodisc^®^ Syringe filters (0.2 μm, 13 mm) were purchased from Pall (Pensacola, FL, USA). Disposable sterilized syringes (1 mL) and needles were purchased from Fenglin Medical Apparatus Corporation Ltd. (Jiangxi, China). Pipette tips (1000 μL, 200 μL and 20 μL) were purchased from Labmed Biotech Corporation (USA). Centrifuge tubes were from KiRGEN (Atlanta, GA, USA). Vials (1.5 mL and 2 mL) were purchased from Waters (Milford, MA, USA).

HPLC-grade methanol, acetonitrile, and water were from Fisher (Pittsburgh, PA, USA). Formic acid (LC/MS grade) was purchased from Merck Millipore (Darmstadt, Germany). ACS grade sodium hydroxide was obtained from Sigma-Aldrich (St. Louis, MO, USA). Distilled water was purchased from Watsons (Guangzhou, China). Lastly, ultrapure water was obtained through Milli-Q purification system (Merck Millipore, Darmstadt, Germany). Other solvents and reagents used were of analytical grade.

### 3.2. Plant Materials

The roots of *M. officinalis* (How.) and *M. citrifolia* (Linn.) were collected from the germplasm repository of tropical medicinal plants, Danzhou City, Ministry of Agriculture, Hainan Province, China, in November and December 2018. *M. officinalis* (How.) was introduced from Diaoluoshan, Lingshui Li Autonomous Count, Hainan Province, China. *M. citrifolia* (Linn.) was introduced from Xisha yongxing island, Hainan Province, China.

The identification of the voucher specimens was authenticated by Maoyuan Wang from the Tropical Crops Genetic Resources Institute, Chinese Academy of Tropical Agricultural Sciences. Specimens were then deposited in the Key Laboratory of Crop Gene Resources and Germplasm Enhancement in Southern China, Ministry of Agriculture, Hainan Haikou, China (No. B20181121 and B20181208). The plant materials were air-dried and ground for the extraction procedure.

### 3.3. Sample Preparation

The air-dried roots of *M. officinalis* and *M. citrifolia* were ground and passed through an 80-mesh sieve. Each powdered sample (1.0 g) was mixed with methanol (50 mL) and then sonicated for 1 h at a temperature of 40 °C. The extract was subjected to centrifugation (12,000 rpm, 10 °C) for 10 min. The supernatant was passed through a 0.2 μm polytetrafluoroethylene (PTFE) syringe filter (VWR Bridgeport, PA, USA) and transferred to a 2 mL transparent vial [19].

### 3.4. UPLC/Q-TOF-MS Detection Conditions

#### 3.4.1. Chromatographic Conditions

The separation was conducted on a Waters ACQUITY UPLC system with an ACQUITY UPLC HSS T3 C18 column and 0.1% aqueous formic acid (A) and acetonitrile with 0.1% formic acid (B) was applied as the solvent system. The flow rate was 0.3 mL/min, and each sample solution was injected 1 μL. The column temperature was controlled at 40 °C. Five injections each were performed for both, sample and the blank control. A linear gradient elution was used as follows: 0–4 min, 3–15% B; 4–12 min, 15–60% B; 12–16 min, 60–80% B; 16–18 min, 80–100% B; 18–21 min, 100–100% B; 21–22 min, 100–3% B; 22–25 min, 3–3% B.

#### 3.4.2. Mass Spectometry Conditions

A Waters Xevo G2 QTOF equipped with Z-Spray electro spray ionization (ESI) source and MassLynx 4.1 was used for MS data acquisition. Negative ionization mode was applied to acquire data under a mass range of 50−1200 Da with a scan time of 0.2 s and detection time of 22 min; furthermore, both low-energy (function 1) and high-energy (function 2) scan functions were used. The collision energy was 6 V for the low-energy scan function, and 10–45 V for the high-energy scan function. The capillary voltages were set at 2.0 kV, and the sampling cone voltage at 40 V. The source and desolvation temperatures were 100 °C and 400 °C, respectively. The desolvation gas flow rate was 600 L/h, while the cone gas flow rate was 50 L/h.

### 3.5. Mass Data Processing and Analysis

Progenesis QI software was utilized to convert the data of UPLC/Q-TOF-MS, i.e.,; then, the significant differential retention time-exact mass (RT-EM) pairs in the S-plots were picked and exported back into Progenesis QI for compound structural elucidation. The resultant data matrices were subsequently exported to EZinfo 2.0 software (MassLynx v4.1, Waters) for PCA (principal component analysis) and an orthogonal partial least squares-discriminant analysis (OPLS-DA) to examine differences in the samples. All variables obtained from UPLC-MS datasets were mean-centered and scaled to Pareto variance before subjection to PCA and OPLS-DA. The OPLS-DA score plots were depicted by the cross-validation parameter R2Y and Q2, which signify the total explained variation for the X matrix and the predictability of the model, respectively. The sum of squares of the PLS weights was evaluated by the value of VIP (variable importance in projection), showing the relative contribution of each X variable in the model. The variables with VIP >3 were considered influential for the separation of samples in the score plots from the PLS-DA analysis [26,27].

### 3.6. Chemical Composition and Biomarker Identification

Multiple approaches were applied to identify chemical components and biomarkers, which include; comparison with retention times, accurate molecular ions, and characteristic fragment ions of reference compounds; reported data of the same compounds in the literature; online Traditional Chinese Medicine (TCM) Chinese [UNIFI1.7]; and ChemSpider [27].

### 3.7. Cell Viability Assay

Cells were planted into 96-well plates. Various concentrations of the extracts (0.4, 1.2, 3.7, 11.1, 33.3 and 100 μg/mL) were added into the culture media for 24 h, 48 h, and 72 h. At 1 h before each time point, a 10 μL volume of CCK-8 reagent was added to each well. Optical density values at 450 nm were read with a microplate reader (Elx808, Bio Tek, Winooski, VT, USA) and analyzed with GraphPad Prism 7.0 software (GraphPad, San Diego, CA, USA).

### 3.8. Cell Colony Formation Assay

Cells were plated into 12-well plates in a triplicate manner. The root extracts of *M. officinalis* and *M. citrifolia* (25, 50, and 100 μg/mL) were added to the plates. Culture media were replaced every third day. After 10 days, the culture medium was discarded, and cells were washed by phosphate-buffered saline (PBS). Then, cells were fixed with 4% paraformaldehyde (Beyotime Biotechnology, Shanghai, China) for 30 min, and stained with crystal violet (Beyotime) for 30 min, followed by a wash with distilled water. The colonies were imaged by a HP scanner and counted under an inverted phase contrast microscope.

### 3.9. Data Analysis

Cell experiments were performed in triplicate, and the results are presented as the mean value ± standard error of the mean (SEM). Data were analyzed using GraphPad Prism 7.0 software (GraphPad, San Diego, CA, USA), and p < 0.05 was considered statistically significant.

## 4. Conclusions

In the present study, a total of 49 compounds were identified from the methanol extract of the root of *M. officinalis* and 39 compounds were identified from the methanol extract of the root of *M. citrifolia* by a UPLC-QTOF-MS/MS analysis. We found 25 ingredients that were present in both plants; they consisted of anthraquinones, triterpene, and iridoid glycosides. Using a QI analysis, we identified 9 components that could be used as chemical markers to distinguish the root extracts of *M. officinalis* from those of *M. citrifolia* introduced from Hainan Province. In addition, we found that *M. officinalis* and *M. citrifolia* exhibited no cell cytotoxicity towards normal cells, and even promoted the proliferation of normal liver cells. Our study suggests that *M. officinalis* and *M. citrifolia roots* have similarities in terms of chemical composition and biological activity. However, further research is needed to explore the feasibility and substitutability of the clinical application of *M. officinalis* and *M. citrifolia*.

## Figures and Tables

**Table 1:** The mass spectrometry data and identified chemical compounds from *M. officinalis* and *M. citrifolia.*

Peak No.	Rt^e^ (min)	Formula	Quasi-Molecular Ion	MS^2^ Ions	Molecular Weight		Mass Error (ppm)	Identification	Type	Source	
					Observed	Theoretical				M. O	M. C
1	1.76	C_16_H_22_O_11_	389.1087 [M − H]^−^	209.0457 137.0610	390.1159	390.1162	−0.7	monotropein	I	√	√
2	2.30	C_16_H_22_O_11_	389.1089 [M − H]^−^	-	390.1162	390.1162	−0.1	diacetylasperulosidic acid	I	√	√
3	3.03	C_16_H_22_O_10_	419.1191 [M + HCOO]^−^	211.0613 123.0454	374.1209	374.1213	−1.0	geniposidic acid	I	√	√
4	3.17	C_18_H_24_O_12_	477.1248 [M + HCOO]^−^	417.1039 371.0983 209.0453 191.0350 165.0559 161.0248 147.0454	432.1266	432.1268	−0.4	isoasperulosidic acid	I	√	√
5	3.67	C_17_H_26_O_11_	451.1454 [M+HCOO]^−^	243.0875	406.1472	406.1475	−0.8	harpagide acetate	I	√	√
6	4.10	C_18_H_24_O_12_	431.1196 [M − H]^−^	371.0974 251.0563 165.0560	432.1269	432.1268	0.3	asperulosidic acid	I	√	√
7	4.78	C_21_H_32_O_15_	523.1666 [M − H]^−^	477.1613 293.0876 233.0669	524.1739	524.1741	−0.4	rehmannioside A	I	√	√
8	4.86	C_18_H_22_O_11_	459.1143 [M + HCOO]^−^	147.0454	414.1161	414.1162	−0.3	asperuloside	I	√	√
9	6.44	C_26_H_28_O_15_	579.1342 [M − H]^−^	413.1078 285.0398 146.9650	580.1415	580.1428	−2.4	3-*O*-primeverose-1,6,8-trihydroxy-2-methyl-anthraquinone	A		√
10	6.84	C_27_H_30_O_15_	593.1507 [M − H]^−^	253.0506	594.1580	594.1585	−0.8	1-*O*-primeverose-3-methoxy-8-hydroxy-2-methylol-anthraquinone	A	√	√
11	6.89	C_27_H_30_O_14_	623.1616 [M + HCOO]^−^	283.0612 253.0506 146.9654	578.1634	578.1636	−0.2	1-*O*-gentiobiose-2-methylol-anthraquinone	A	√	√
12	7.21	C_22_H_22_O_10_	491.1195 [M + HCOO]^−^	283.0613	446.1213	446.1213	−0.1	1-*O*-β-d-glycopyranosyl-8-methoxy-emodin	A		√
13	7.23	C_28_H_32_O_14_	637.1771 [M + HCOO]^−^	547.1449 307.0609 297.0763 283.0613	592.1789	592.1792	−0.4	1-*O*-primeverose-3,8-dimethoxy-2-methyl-anthraquinone	A		√
14	7.25	C_27_H_30_O_15_	593.1510 [M − H]^−^	269.0456 251.0350 146.9655	594.1583	594.1585	−0.3	1-*O*-gentiobiose-emodin	A	√	
15	7.27	C_26_H_28_O_14_	563.1405 [M − H]^−^	269.0457 251.0352 237.0557	564.1478	564.1479	−0.2	1-*O*-primeverose-aloeemodin	A	√	√
16	7.34	C_27_H_30_O_14_	577.1561 [M − H]^−^	253.0504	578.1634	578.1636	−0.3	3-*O*-gentiobiose -1-hydroxy-2-methyl-anthraquinone	A	√	√
17	7.40	C_27_H_30_O_14_	623.1617[M + HCOO]^−^	269.0455	578.1635	578.1636	−0.1	8−*O*-primeverose-1-methoxy-3-hydroxy-2- methyl -anthraquinone	A	√	
18	7.53	C_27_H_30_O_14_	577.1559 [M − H]^−^	283.0619	578.1632	578.1636	−0.7	3-*O*-primeverose-8-methoxy-1-hydroxy- 2- methyl -anthraquinone	A	√	
19	7.57	C_26_H_28_O_14_	609.1461 [M + HCOO]^−^	415.1024 269.0457	564.1479	564.1479	0.0	8-*O*-primeverose-1,3-dihydroxy-2-methyl -anthraquinone	A	√	
20	7.61	C_27_H_30_O_13_	607.1663 [M + HCOO]^−^	253.0505	562.1681	562.1686	−0.8	1-*O*-gentiobiose -3-hydroxy- 2-methyl -anthraquinone	A	√	√
21	7.65	C_21_H_20_O_10_	431.0977 [M − H]^−^	269.0455 251.0347	432.1049	432.1057	−1.7	emodin 1-*O*-β-d-glycopyranosyl	A		√
22	7.74	C_26_H_28_O_14_	563.1404 [M − H]^−^	269.0456 251.0348 223.0407	564.1477	564.1479	−0.4	1-*O*-primeverose-3,8-dihydroxy-2-methyl -anthraquinone	A	√	√
23	7.75	C_28_H_32_O_15_	607.1665 [M − H]^−^	563.1403 283.0617 267.0661 251.0348	608.1738	608.1741	−0.6	1-*O*-gentiobiose-8-methoxy-aloeemodin	A		√
24	7.80	C_27_H_30_O_14_	577.1556 [M − H]^−^	283.0618	578.1629	578.1636	−1.2	1-*O*-primeverose-8-methoxy-3-hydroxy-2-methyl-anthraquinone	A	√	
25	7.87	C_27_H_30_O_15_	639.1558 [M + HCOO]^−^	299.0562	594.1576	594.1585	−1.3	1-*O*-primeverose-3,4-dimethoxy-2-hydroxy-anthraquinone	A	√	
26	7.89	C_26_H_28_O_14_	563.1405 [M − H]^−^	269.0457	564.1478	564.1479	−0.2	3-*O*-primeverose-1,8-dihydroxy-2-methyl -anthraquinone	A	√	
27	7.93	C_27_H_30_O_15_	593.1502 [M − H]^−^	547.1452 283.0608 285.0405 251.0347	594.1574	594.1585	−1.7	1-*O*-primeverose-2- methylol-3-hydroxy-8-methoxy-anthraquinone	A		√
28	8.08	C_22_H_22_O_9_	475.1243 [M + HCOO]^−^	267.0658 253.0506	430.1261	430.1264	−0.5	1-*O*-β-d-glycopyranosylrubiadin3-methyl ether	A		√
29	8.10	C_26_H_28_O_13_	547.1451 [M − H]^−^	253.0506	548.1524	548.1530	−1.1	1-*O*-primeverose-2-methylol-anthraquinone	A		√
30	8.15	C_21_H_20_O_10_	431.0977 [M−H]^−^	269.0456	432.1050	432.1057	−1.5	1-*O*-β-d-glycopyranosyl-emodin	A		√
31	8.25	C_27_H_30_O_15_	593.1514 [M − H]^−^	269.0457 265.0501	594.1587	594.1585	0.4	1-*O*-gentiobiose-emodin	A	√	√
32	8.37	C_28_H_32_O_15_	653.1720 [M + HCOO]^−^	313.0717 298.0478	608.1738	608.1741	−0.6	1-*O*-primeverose-8-hydroxy-ibericin	A		√
33	8.40	C_27_H_30_O_15_	593.1512 [M − H]^−^	547.1455 431.0981 269.0455	594.1585	594.1585	0.1	8-*O*-gentiobiose-emodin	A	√	√
34	8.59	C_26_H_28_O_13_	593.1513 [M + HCOO]^−^	253.0508 146.9655	548.1531	548.1530	0.2	1-*O*-primeverose-rubiadin	A	√	√
35	8.67	C_26_H_28_O_14_	563.1407 [M − H]^−^	-	564.1479	564.1479	0.0	1-*O*-primeverose-emodin	A	√	√
36	8.81	C_26_H_28_O_15_	579.1353 [M − H]^−^	285.0403	580.1426	580.1428	−0.4	1-O-primeverose-2-methyl-3,6,8-trihydroxy-anthraquinone	A		√
37	8.84	C_30_H_48_O_7_	519.3323 [M − H]^−^	489.3215	520.3396	520.3400	−0.8	(3α)-3,19,20,24,30-pentahydroxyurs-12-en-28-oic acid	T	√	
38	8.98	C_15_H_10_O_4_	253.0509 [M − H]^−^	223.0402	254.0582	254.0579	1.1	1-hydroxy-2-methylol-anthraquinone	A	√	√
39	9.00	C_15_H_10_O_5_	269.0457 [M − H]^−^	-	270.0530	270.0528	0.5	aloe-emodin	A		√
40	9.07	C_15_H_10_O_3_	283.0614 [M + HCOO]^−^	-	238.0632	238.0630	0.9	1-methyl-3-hydroxy-anthraquinone	A	√	√
41	9.08	C_15_H_10_O_4_	253.0510 [M − H]^−^	-	254.0582	254.0579	1.3	rubiadin	A	√	√
42	9.79	C_19_H_16_O_7_	401.0877 [M + HCOO]^-^	283.0607 269.0452	356.0895	356.0896	−0.3	fridamycin E	A	√	√
43	10.07	C_15_H_10_O_5_	269.0458 [M − H]^−^	251.0353	270.0531	270.0528	0.9	emodin	A	√	√
44	10.27	C_15_H_10_O_4_	253.0511 [M − H]^−^	238.0275	254.0584	254.0579	1.8	3-hydroxy-2-methylol-anthraquinone	A	√	
45	10.46	C_30_H_46_O_6_	501.3222 [M − H]^−^	483.3111 457.3323	502.3295	502.3294	0.1	(3α,19β)-3,19,23-trihydroxy-6-oxoolean-12-en-28-oic acid	T	√	
46	10.53	C_15_H_10_O_3_	283.0614 [M + HCOO]^−^	-	238.0632	238.0630	0.7	1-hydroxy-2-methyl-anthraquinone	A		√
47	10.82	C_15_H_10_O_5_	269.0457 [M − H]^−^	-	270.0530	270.0528	0.6	1,3-dihydroxy-2-methoxy-anthraquinone	A		√
48	10.97	C_30_H_48_O_6_	549.3426 [M + HCOO]^-^	485.3259	504.3444	504.3451	−1.2	2,3,6,23-tetrahydroxyurs-12-en-28-oic acid	T	√	
49	11.00	C_16_H_12_O_4_	267.0658 [M − H]^-^	252.0424 224.0470	268.0731	268.0736	−1.7	rubiadin 3-methyl ether	A	√	
50	11.06	C_30_H_48_O_6_	503.3377 [M − H]^-^	485.3268	504.3449	504.3451	−0.3	2,3,19,23-tetrahydroxyolean-12-en-28-oic acid	T	√	
51	11.14	C_16_H_12_O_4_	267.0665 [M − H]^-^	224.0476	268.0738	268.0736	0.7	rubiadin 1-methyl ether	A	√	√
52	11.97	C_30_H_48_O_5_	487.3424 [M − H]^-^	469.3322 437.3048	488.3497	488.3502	−1.0	(3β,6α)-3,6,24-trihydroxyolean-12-en-28-oic acid	T	√	
53	12.26	C_30_H_48_O_5_	485.3273 [M − H]^-^	467.3164 441.3372 423.3266	486.3346	486.3345	0.2	(3β,6α)-3,6,23-trihydroxyursa-12,19(29)-dien-28-oic acid	T	√	
54	12.91	C_30_H_48_O_5_	487.3433 [M − H]^-^	469.3324 443.3528 425.3422	488.3506	488.3502	0.9	(2α,3β,19α)-2,3,19-trihydroxyolean-12-en-28-oic acid	T	√	
55	13.01	C_30_H_48_O_5_	487.3429 [M − H]^-^	441.3368 425.3424 409.3109	488.3501	488.3502	−0.1	(2α,3α)-2,3,24-trihydroxyolean-18-en-28-oic acid	T	√	
56	13.06	C_17_H_14_O_5_	297.0766 [M − H]^-^	251.0348	298.0839	298.08412	−0.8	ibericin	A	√	
57	13.11	C_30_H_48_O_5_	487.3425 [M − H]^-^	441.3368 425.3424 409.3109	488.3498	488.3502	−0.8	(3α)-3,19,23-trihydroxyurs-12-en-28-oic acid	T	√	
58	13.26	C_30_H_48_O_5_	533.3475 [M+HCOO]^-^	441.3364	488.3493	488.3502	−1.7	2,3,23-trihydroxyurs-12-en-28-oic acid	T	√	
59	13.53	C_33_H_50_O_8_	573.3427 [M − H]^-^	-	574.3499	574.3506	−1.1	butyl (3β,7β,12β)-12-acetoxy-3,7-dihydroxy-4,4,14-trimethyl-11,15-dioxochol-8-en-24-oate	T	√	
60	14.30	C_31_H_50_O_6_	517.3527 [M − H]^-^	471.3470	518.3600	518.3607	−1.4	(1α,3β,9β)-24-hydroperoxy-1,3-dihydroxy-5-methyl-9,19-cyclolanost-25-en-28-oic acid	T	√	
61	14.61	C_30_H_48_O_4_	471.3474 [M − H]^-^	453.3369 427.3583	472.3547	472.3553	−1.2	hederagenin	T	√	
62	15.95	C_30_H_48_O_4_	517.3528 [M + HCOO]^-^	423.3266	472.3546	472.3553	−1.3	maslinic acid	T	√	
63	19.52	C_29_H_44_O_2_	423.3264 [M − H]^-^	325.0718	424.3337	424.3341	−1.1	camphoratin H	T	√	√

I: iridoid glycosides; A: anthraquinones; T: triterpenes. M. O: *M. officinalis; M. C: M. citrifolia.*

## Supplementary Materials

The following are available online. Figure S1: The propose fragmentation pathways of anthraquinones; Figure S2: The propose fragmentation pathways of anthraquinones.
