# Supplementary material for: Comparison of Anthraquinones, Iridoid Glycosides and Triterpenoids in Morinda officinalis and Morinda citrifolia Using UPLC/Q-TOF-MS and Multivariate Statistical Analysis
Source: Molecules. 2019 Dec 31;25(1):160. doi: 10.3390/molecules25010160 (PMC6983063; doi:10.3390/molecules25010160)
Supplement: Supplementary file 1 [file molecules-25-00160-s001.pdf]

Supplementary materials

Comparison of anthraquinones, iridoid glycosides and triterpenoids in Morinda officinalis and M. citrifolia using UPLC/Q-TOF-MS and Multivariate Statistical Analysis

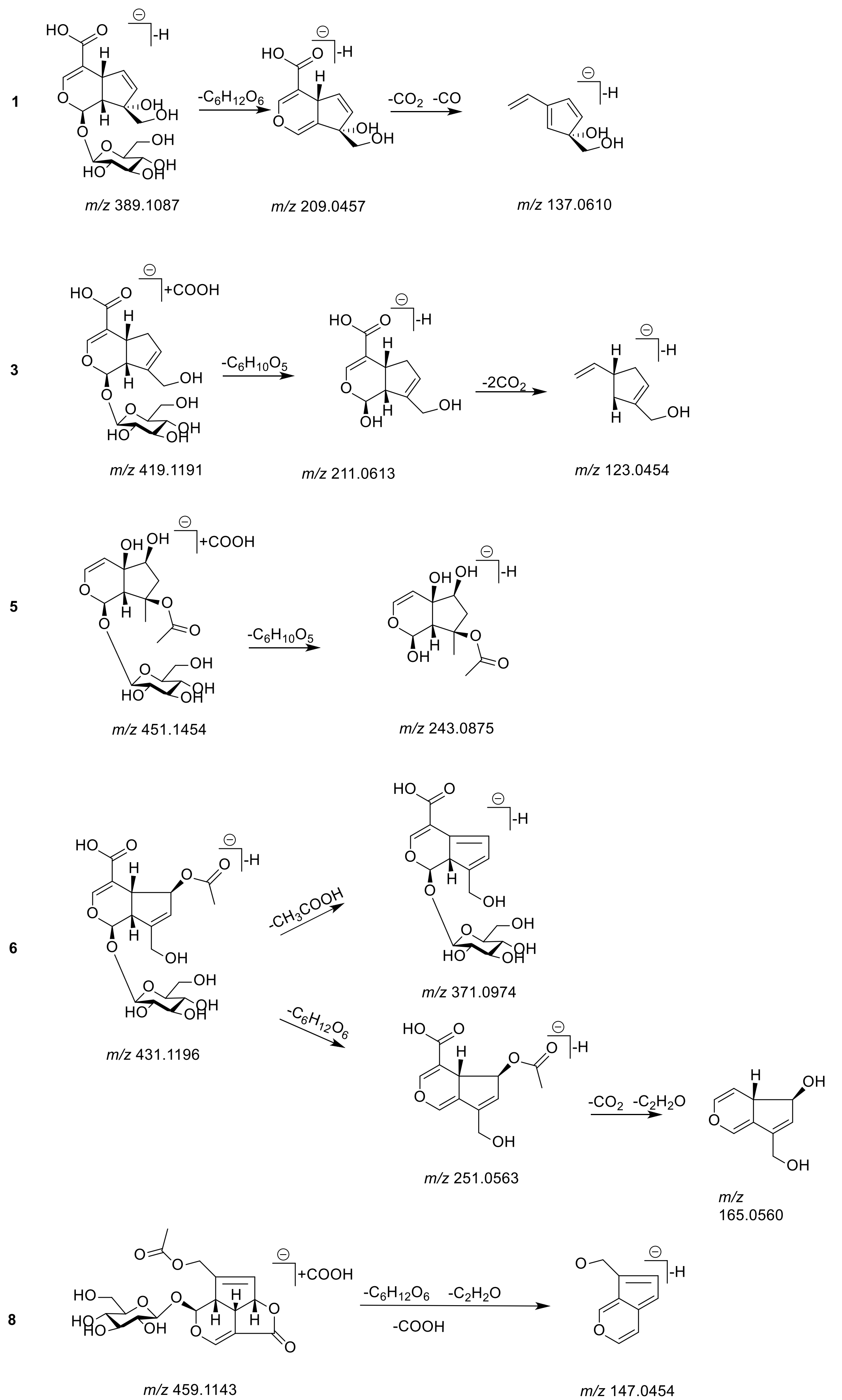

Fig. S1 The propose fragmentation pathways of iridoid glycosides.

9

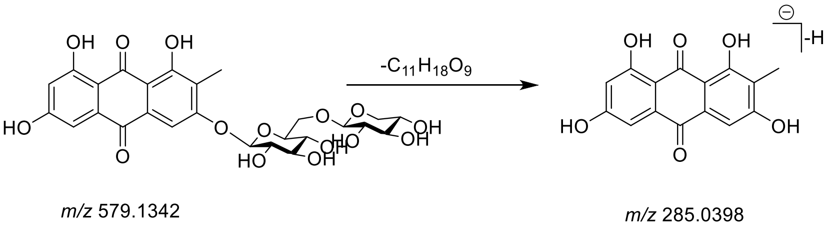
-$C_{11}H_{18}O_9$
m/z 579.1342
m/z 285.0398

10

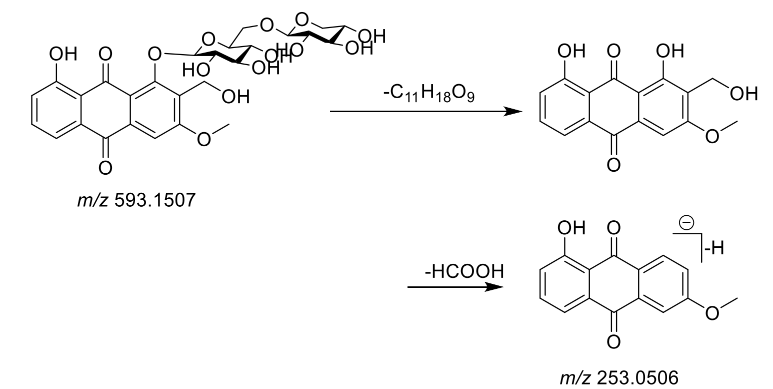
-$C_{11}H_{18}O_9$
m/z 593.1507
-HCOOH
m/z 253.0506

11

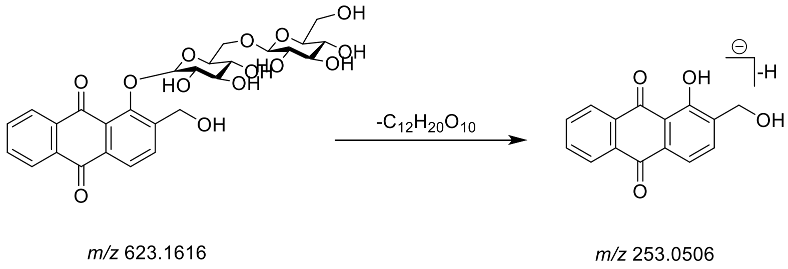
-$C_{12}H_{20}O_{10}$
m/z 623.1616
m/z 253.0506

12

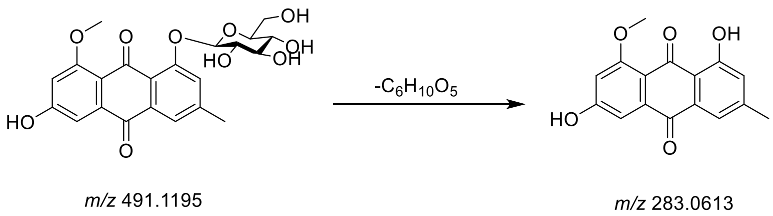
-$C_6H_{10}O_5$
m/z 491.1195
m/z 283.0613

13

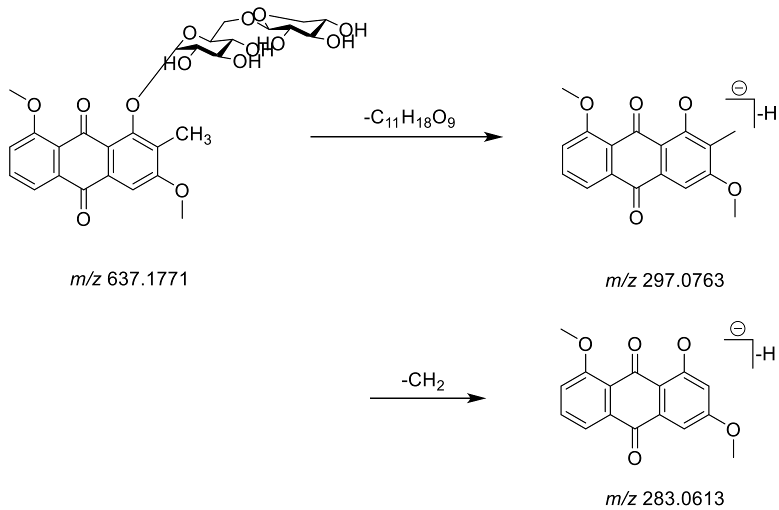
-$C_{11}H_{18}O_9$
m/z 637.1771
m/z 297.0763
-CH_2
m/z 283.0613

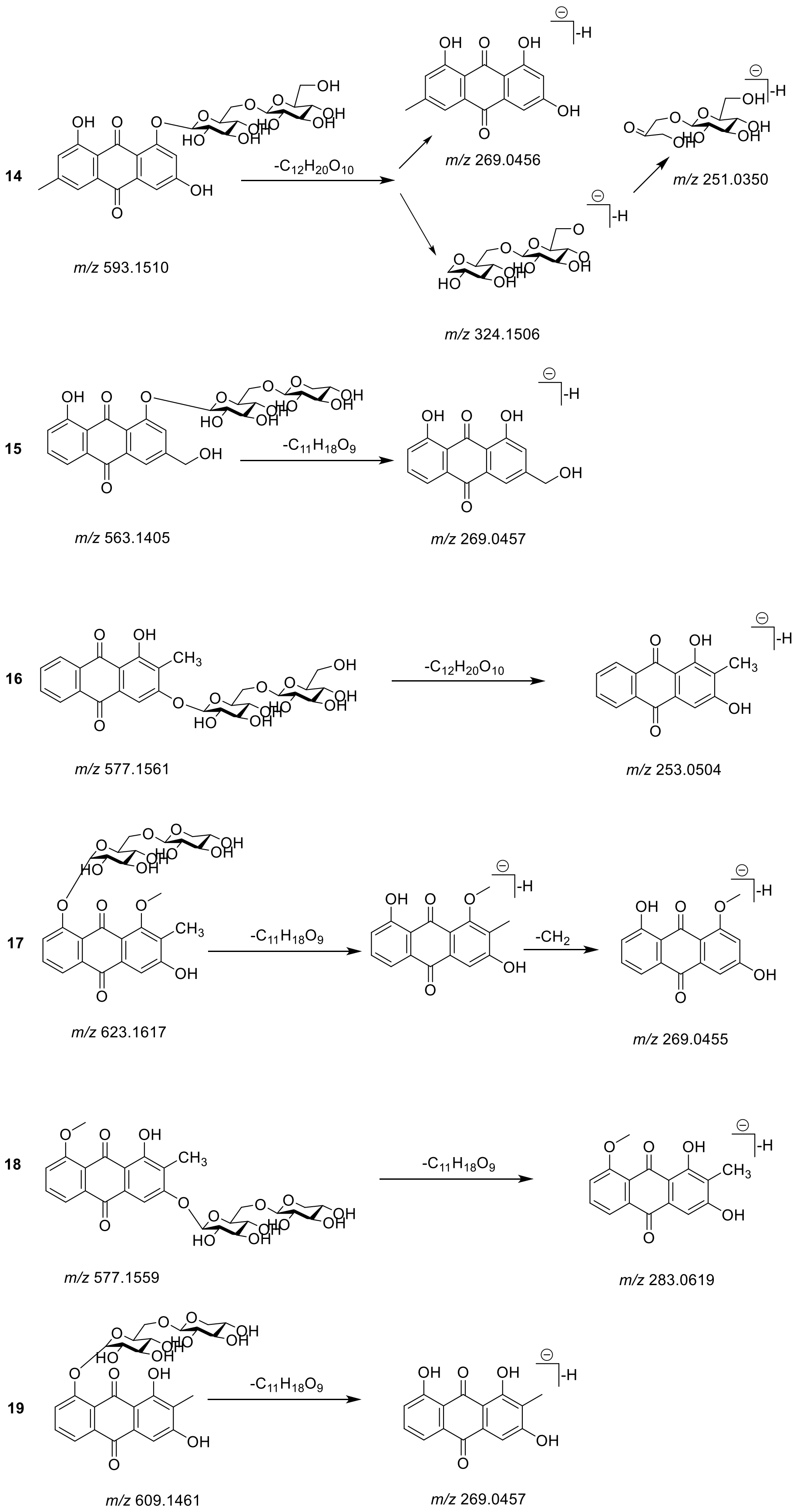

Fig. S2 The propose fragmentation pathways of anthraquinones.
